# Genotoxicity of Nanomaterials: Advanced In Vitro Models and High Throughput Methods for Human Hazard Assessment—A Review

**DOI:** 10.3390/nano10101911

**Published:** 2020-09-25

**Authors:** Yvonne Kohl, Elise Rundén-Pran, Espen Mariussen, Michelle Hesler, Naouale El Yamani, Eleonora Marta Longhin, Maria Dusinska

**Affiliations:** 1 Fraunhofer Institute for Biomedical Engineering IBMT, 66280 Sulzbach, Germany; michelle.hesler@ibmt.fraunhofer.de; 2 Health Effects Laboratory, NILU-Norwegian Institute for Air Research, 2007 Kjeller, Norway; erp@nilu.no (E.R.-P.); ema@nilu.no (E.M.); ney@nilu.no (N.E.Y.); eml@nilu.no (E.M.L.); mdu@nilu.no (M.D.)

**Keywords:** nanomaterials, genotoxicity, in vitro 3D models, high throughput methods, miniaturization

## Abstract

Changes in the genetic material can lead to serious human health defects, as mutations in somatic cells may cause cancer and can contribute to other chronic diseases. Genotoxic events can appear at both the DNA, chromosomal or (during mitosis) whole genome level. The study of mechanisms leading to genotoxicity is crucially important, as well as the detection of potentially genotoxic compounds. We consider the current state of the art and describe here the main endpoints applied in standard human in vitro models as well as new advanced 3D models that are closer to the in vivo situation. We performed a literature review of in vitro studies published from 2000–2020 (August) dedicated to the genotoxicity of nanomaterials (NMs) in new models. Methods suitable for detection of genotoxicity of NMs will be presented with a focus on advances in miniaturization, organ-on-a-chip and high throughput methods.

## 1. Introduction

Nanomaterials (NMs) are materials with unique properties and size with at least one dimension between 1 and 100 nm, as defined by the European Commission (Regulation (EC) No. 1907/2006) [1]. They have already been in use for several decades, and are being utilized in almost every industrial sector. Due to constantly growing commercial applications and their presence in the majority of consumer products, NMs are being extensively investigated for their safety. A main concern with NM exposure is their genotoxic potential. Genotoxicity describes the property of chemical or physical agents that are able to alter the genetic information. Genotoxic events can be transient (repairable damage), or can lead to permanent changes (mutations) in the amount or structure of the genetic material in a cell. When a mutation is present in a germ cell, it is inherited to the next generation, and can cause a genetic disorder. In somatic cells, a mutation in a critical gene may lead to cancer. Thus, every mutagen is considered to be potentially carcinogenic. 

A strategy for genotoxicity testing needs to cover all necessary endpoints: gene mutations, chromosomal aberrations (clastogenicity) and larger genome damage leading to aneuploidy. All these endpoints can be covered by the so-called two-test strategy, covering gene mutations, chromosomal aberrations (clastogenicity) and changes in number of chromosomes (aneuploidy). Because of the size-related special physicochemical properties of NMs, many standard test methods are not suitable for the toxicity testing of NMs or the test protocols have to be optimized. For assessing gene mutations caused by NMs, the Ames test is not suitable owing to the small size of bacteria (comparable with NMs) as well as the bacterial cell wall which is different from the human/mammalian cell membrane [2,3,4]. Thus, in the test strategy the mammalian gene mutation test is recommended together with the micronucleus assay [4,5]. Additionally, many other assays exist addressing intermediate endpoints, such as various types of DNA damage and novel markers as omics or epigenetic changes [6]. These markers can give supporting evidence about the safety of tested compounds, including NMs [7].

Standard in vitro genotoxicity tests are typically based on a single cell type. However, a key observation in in vivo genotoxicity studies conducted with NMs is that the permanent changes induced in DNA are often the result of secondary genotoxicity associated with inflammation [8,9,10,11]. Thus, there has been a strong effort in recent years to develop more complex, in vivo-like in vitro models based on 3D structures either of a single cell type or as co-cultures of two or more cell types. The application of these models in toxicology is believed to provide reliable data that are more relevant for evaluating genotoxicity in humans than standard 2D models. Several of these models are already well advanced, and new protocols have been established for example for skin, lung and liver tissue models [4,6]. The advanced 3D models are technically more difficult to perform and more time-consuming; however, they can better address exposure scenarios, such as lung models reflecting inhalation exposure, skin models for dermal exposure, and liver models for systemic or oral exposure. Another advantage of such advanced models is that most 3D models are based on human cells, enhancing the relevance of the results for assessing potential human genotoxicity. As there are huge numbers of NMs to be tested, research in in vitro toxicology focuses on miniaturization and development of robust high throughput methods. An overview of standard and novel genotoxicity tests, advanced models and high throughput methods for application with NMs is provided below [12,13,14].

## 2. Methods

Our approach was to describe the main endpoints applied in new advanced in vitro 3D models, organ-on-chip and high throughput methods suitable for detection of genotoxicity of NMs. An electronic literature search was conducted through PubMed databases to identify articles dealing with genotoxicity of NMs and advanced in vitro models and high throughput methods from year 2000–2020 (August). The following keyword combinations were used: “genotoxicity & 3D in vitro model”, “genotoxicity & advanced in vitro model”, “genotoxicity & high throughput”, “genotoxicity & high throughput & nanomaterials“, “genotoxicity & high throughput & nanoparticles”, “genotoxicity & organ on chip”, “genotoxicity & 3D models & nanoparticles”, “genotoxicity & 3D models & nanomaterials”. As publication type, both research articles as well as reviews were considered. 

## 3. Results of Literature Search

**Figure 1:**
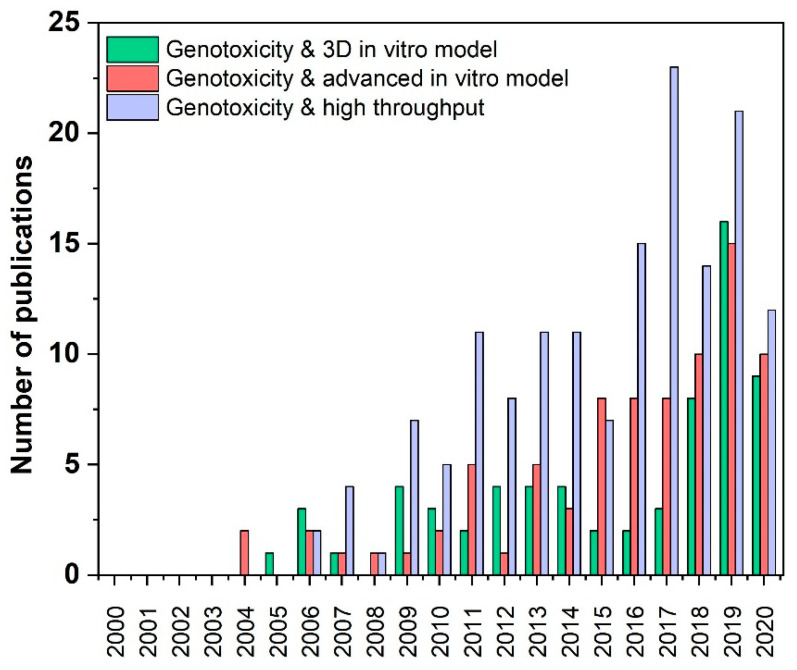
Number of publications during the last 20 years in the field of advanced in vitro 3D models, organ-on-chip and high throughput methods suitable for detection of genotoxicity of nanomaterials. An electronic literature search was conducted through PubMed databases to identify articles dealing with genotoxicity of nanomaterials and advanced in vitro models and high throughput methods from year 2000–2020 (August). The number of publications is assigned to the individual years.

The literature search resulted in 59 papers for “genotoxicity & 3D in vitro model”, 73 papers for “genotoxicity & advanced in vitro model”, 134 papers for “genotoxicity & high throughput”, eight papers for “genotoxicity & high throughput & nanomaterials” [15,16,17,18,19,20,21] and six papers for “genotoxicity & high throughput & nanoparticles” (4 are the same as for “genotoxicity & high throughput & nanomaterials”) [16,17,18,19,21,22], seven papers for “genotoxicity & organ on chip“ [23,24,25,26,27,28,29], seven papers for “genotoxicity & 3D models & nanoparticles” [30,31,32,33,34,35,36], 11 papers for “genotoxicity & 3D models & nanomaterials” [30,31,33,36,37,38,39,40,41] for the period of 20 years (2000–2020), whereby the year 2020 was considered only from January to August. (Table 1). 265 (88.0%) out the overall 301 studies have been published in the last ten years and 91 (30.2%) out the overall studies in 2019 and 2020 (Table 1). The accelerated publication of papers on these topics over the last few years clearly shows that the importance of the fields of research is increasing (Figure 1). This is partly due to the development of alternative methods to animal testing, as required by REACH (Registration, Evaluation, Authorisation and Restriction of Chemicals) [42], and to the ever-increasing implementation of the 3Rs (Replace, Reduce, Refine) principle [43].

If one differentiates again all identified publications on the topic of “genotoxicity & 3D models & nanomaterials/nanoparticles/organ on chip”, with regard to review articles and research studies and studies mentioned twice, it becomes clear that much less is known and so far only ten studies have been published dealing with in vitro 3D models and organ-on-chip systems for the detection of genotoxicity of NMs. A detailed overview of the study designs is shown in Table 2.

## 4. General Mechanisms of Genotoxicity

The genotoxicity of NMs and mechanisms leading to transient or permanent genetic changes have been intensively investigated [8,9,10,11,12,13]. Recent studies show that NM genotoxicity can result from two main mechanisms; primary (direct or indirect) or secondary genotoxicity. For certain NMs, one of these mechanisms might apply; however, for some NMs both mechanisms can occur simultaneously after NM exposure. 

Primary direct genotoxicity is caused by direct interaction of NMs with the genome, and requires physical contact of NMs with DNA in the nucleus. This interaction could lead to DNA damage such as DNA breaks and other DNA lesions, large DNA malformation, or chromosomal damage. The primary indirect mechanism arises from NM-induced reactive oxygen species (ROS), or from toxic ions released from dissolution of NMs and production of intracellular ROS via the Fenton-type reaction [5,12]. Oxidative stress is considered a key mechanism in primary indirect genotoxicity. Free radicals may interact with cellular biomolecules including DNA, leading to potentially serious consequences. ROS may attack the DNA causing purine or pyrimidine oxidation lesions and strand breaks. The damage can be repaired but can also result in gene mutations and even larger chromosomal damage. NMs can promote DNA damage also via other molecules that either have the capacity to interact with DNA or interfere with DNA replication and cell division. NMs can interact for example with protein kinases responsible for regulation of cell cycle events, such as DNA replication and cell division [11].

For understanding whether primary genotoxicity is direct or indirect, it is important to study the mechanism of uptake and whether NMs can enter the nucleus. The smallest NMs (with a size of only a few nm) could penetrate the nucleus via nuclear pores. However, some studies show the presence of larger NMs in the nucleus, indicating that there could be other pathways for nuclear uptake, for example intracellular processes resembling endocytosis [45]. NMs can enter the nucleus also during mitosis when the nuclear membrane is dissolved. Once in the nucleus, NMs might directly interact with DNA, or with DNA organized in chromosomal structures, depending on the stage of the cell cycle (direct genotoxicity). During interphase, NMs could interact or bind with DNA molecules and interfere with DNA replication and transcription of DNA into RNA. NMs can also interfere with the spindle apparatus, the centrioles and associated proteins and disturb the process of mitosis, thus leading to the formation of micronuclei that could be manifested as clastogenicity or aneugenicity [46].

Secondary genotoxicity is considered to be the main mechanism of genotoxicity of NMs. It is mediated via ROS produced by inflammatory cells. NMs can trigger an oxidative burst caused by activation of phagocytes. This inflammation is an initial defense mechanism against invasion of microorganisms or foreign materials, involved in clearance. In case clearance of e.g., inhaled NMs fails, it can cause a chronic immune cell response. This type of secondary genotoxicity is not possible to study with standard in vitro approaches, and has until recently been investigated only in vivo following chronic inflammation caused by activation of immune cells, such as macrophages or neutrophils [8,47]. An in vitro approach to studying secondary genotoxicity has many challenges, as standard monoculture systems cannot be used to assess the ability of NMs to induce genotoxicity via inflammation. Advances in new models thus focus on co-culture of target cells with cells from the immune system.

Several studies show that genotoxicity of NMs not only depends on dose and exposure duration, but also on their size, surface properties, chemical composition as well as on shape [48,49,50,51]. 

Huk et al. investigated three silver (Ag) NMs with same surface properties but different sizes and found that Ag NMs genotoxicity depends on NM size [52]. Comparative in vitro genotoxicity studies of zinc oxide (ZnO) nano- and macroparticles cells also came to the conclusion that size matters [53]. Guo et al. summarized their studies, that coatings have less effect on the relative genotoxicity of Ag NMs than the particles size [54]. With both the micronucleus and mouse lymphoma assay, the smaller the Ag NMs, the greater the cyto- and genotoxicity [54]. Also other studies investigated the contribution of nano- and micro-sized fractions (titanium dioxide (TiO_2_) [55], gold (Au) [56], cobalt chrome [57], polystyrene [58], silica [59], Ag [54,60,61], Kaolin [62], ZnO [32], copper (Co) [63]. Using the mini-gel comet assay Lebedová et al. studied the size-dependent genotoxicity of Ag, Au and platinum (Pt) NMs [64]. The authors detected size- and material-dependent DNA strand breaks [64].

In a study with Ag NMs of same size the authors found that genotoxicity and mutagenicity of nano-Ag depends on their surface properties and charge [65]. Similarly Gabelova et al. showed that surface chemistry affects genotoxicity of iron oxide NMs [66]. 

Another study compared the effect of size and surface functionalization of Au NMs on genotoxicity [67]. Two core sizes (5 and 20 nm) and three functionalizations (negative, positive, and neutral surface charges) were examined by Vales et al. [67]. Surface charge-dependent effects have been identified [67]. Further studies found that silica oxide (SiO_2_)-coated nanosized TiO_2_ induced less DNA damage than uncoated [68]. This finding may be associated with abilities that reduce the formation of free radicals mediated by TiO_2_ NMs [69], change NMs’ agglomeration stage [70] and influence the interaction with biological components [71]. A relationship between genotoxicity and surface composition has also been demonstrated for many other metal and metal oxide particles [61,63,71,72,73,74,75] as well as for biopolymer particles [76].

Shape-dependent genotoxicity has also been studied in vitro [77,78]. Different shapes (bipyramids, rods, platelets) of TiO_2_ NMs have been compared to commercial TiO_2_ NMs [78]. Also sphere- and rod-shaped mesoporous SiO_2_ NMs have been screened regarding their genotoxic potential [77]. 

Besides shape, surface properties and size, also the chemical composition plays an important role in NM genotoxicity [61,79]. Studies on genotoxicity of metal NMs (Zn, TiO_2_, Ag, iron oxide, etc.) suggest this [61,79]. 

The interrelationship between particle shape, size, chemical composition and toxicological effects has been explored by Yang et al. [80]. Compared with ZnO NMs, carbon nanotubes were moderately cytotoxic but induced more DNA damage determined by the comet assay [80]. This comparative study demonstrates that chemical composition plays a major role in cytotoxicity and the size in genotoxicity [80]. 

Among different types of metal or metal oxide nanoparticles, the genotoxic potential of carbon-based materials such as SWCNT [81], multi wall carbon nanotube (MWCN) [82,83] or graphene [84,85,86], have also been evaluated in vitro. High-aspect-ratio MWCNTs were found to be more toxic than the low-aspect-ratio MWCNTs [83]. Studies on interactions of graphene oxide and graphene nanoplatelets with the in vitro intestinal barrier model resulted in no oxidative damage induction [87]. Whereas MWCNTs induced significant DNA damage [88,89], an increase in 8 nitroguanine [88,90] and an increase in micronucleused cells [88,91].

Transformation processes in the environment, including chemical, physical and biological processes could affect the toxic potential of NMs. So called aging processes may change the surface properties as well as chemical composition and size of the NMs. Few studies are available investigating the effect of aging on the genotoxic potential of NMs [92,93,94,95,96,97] assuming an influence on genotoxicity, but too little data are available to be able to conclude.

## 5. Nanomaterials–Characteristics in Cell Culture Media and Assay Interference

NMs can be divided into broad categories of materials, such as metals (silver, gold, copper), metal oxides (titanium dioxide, iron oxide, zinc oxide), carbon based NMs (single- or multi-walled carbon nano tubes), different types of polymers and further advanced NMs such as complex, hybrid, multi-component or multi-structure NMs. NMs not only vary in their chemical composition, but also in size, size distribution, shape, surface characteristics, surface area etc. [98]. In vitro studies between different laboratories often contradict one another, due to an inadequate NM characterization under the study conditions. For the most cellular assays, NMs stock solutions are diluted in cell culture medium (CCM). In a second step this suspension is applied to the cultured cells in vitro. When NMs are suspended in CCM or other biological fluids, they change their particle surface, the associated particle-particle and particle-cell interactions [99,100]. This is dependent on the characteristics of the primary NM (composition, size, shape, surface chemistry) and the properties of the surrounding fluid (pH, ionic strength, protein content, temperature) [16,17]. CCM containing fetal bovine serum leads to a formation of so-called protein coronas, which change the physicochemical nature and toxicological behavior of the NMs so enormously, that they cannot be compared any longer to untreated NM stock solutions [99]. Thus, the characterization of NMs under test conditions is indispensable to understand their impact on cellular systems and to interpret the test result accurately [101,102,103]. Typically, the size and size distribution of NMs are determined by light scattering techniques such as dynamic light scattering (DLS) and nanoparticle tracking analysis (NTA). On the other hand, shape and size (distribution) can be directly visualized by microscopic techniques, such as transmission electron microscopy (TEM), scanning electron microscopy (SEM) and atomic force microscopy (AFM) [104]. The surface charge of NMs can be characterized by the determination of the Zeta potential, which is a method to measure the electrostatic potential at the electrical double layer, which surrounds NMs in suspension [105]. Field flow fractionation (FFF) is a powerful sizing technique and in combination with UV- and multi-angle light scattering (MALS)-detectors it is suitable for separation of different particle sizes in a low concentration range. When combined with inductively coupled plasma-mass spectrometry (ICP–MS), this technique allows determination of the (ion) composition and concentration of NM test solutions [106]. Moreover, characterization regarding interference of NMs with the applied assay system is highly recommended. It has been shown that some NMs interfere with fluorescence/absorption in widely used assays such as alamarBlue™, Neutral Red or WST-1, which can lead to false positive cytotoxicity results unless interference test is included in the test [107,108].

## 6. Cytotoxicity Testing before Genotoxicity Studies

Before genotoxicity testing can be performed, it is necessary to know the cytotoxicity of the tested NMs and to establish the LC_50_ (lethal concentration- at which 50% of cells will die) in order to choose the appropriate range of concentrations before genotoxicity testing. The dose range depends on the type of genotoxicity test applied to the NMs. For the gene mutation and micronucleus assays, the concentration range should cover non-cytotoxic concentrations and up to 50 ± 5% cell death. For assays that are detecting DNA breaks, NMs are tested only at non-cytotoxic concentrations, as DNA breaks can also be induced indirectly as a consequence of cytotoxicity.

DNA fragmentation is a step in the cell signaling cascade for induction of apoptotic cell death, downstream of caspase 3 activation and cleavage and activation of the DNA fragmentation factor DFF, and many apoptotic assays are based on detection of DNA fragmentation [109]. As DNA breaks are coupled to the cell death process, false positive results and classification of cytotoxic compounds as genotoxic can occur if cytotoxicity is not an integrated part of the genotoxicity testing. However, there is no consensus when it comes to the threshold value for cytotoxicity. Strictly, cytotoxicity is stated from 80% viability (20% cytotoxicity compared to negative control), but others consider that the cut-off for cytotoxicity in genotoxicity testing should be as low as around 50% viability [110,111].

Cytotoxicity can be tested by different assays [112,113]. The most significant and meaningful tests are based on the ability of cells to proliferate or to survive and form colonies, i.e., viability. These tests are interference-free, and the read-out is based directly on cell survival. The plating efficiency, relative growth activity and colony forming efficiency assays contain all previously described characteristics [22]. Plating efficiency and relative growth are integrated parts of OECD Test Guidelines 476 and 490 [22] (for mammalian gene mutation tests (OECDTG 476, 1997; OECD TG 490, 2015) [114,115]. Alternative measures of cytotoxicity (rather than viability) can be based on membrane integrity and application of membrane impermeable dyes, allowing staining only of cells with damaged cell membrane, such as the Trypan blue exclusion test. Another common method is dual staining with the fluorescent dyes propidium iodide for dead cells and fluorescein diacetate for live cells, and analysis by microscopy or flow cytometer. Dead or dying cells can be detected by leakage of intracellular substances, as lactate dehydrogenase (LDH), or by other colorimetric or fluorometric methods, such as staining with dyes requiring metabolic activation for detection. A common and convenient test is the alamarBlue™ assay, based on reduction of Resazurin to the highly fluorescent Resorufin, or the similar MTT test. Results of cytotoxicity assays that are not based on cell survival should be interpreted with caution; the effects they measure may be reversible, such as damage to the membrane, and so might not indicate cell death. It is important, when selecting a cytotoxicity test for application in genotoxicity testing, that the same exposure conditions should apply to both tests, and that the tests should be performed in parallel. If genotoxicity is measured after, e.g., three and 24 h exposure, as is common for the comet assay, then cytotoxicity should also be measured after three and 24 h’ exposure. The alamarBlue™ assay is a convenient and reliable cytotoxicity assay to be used in combination with the comet assay, which has been tested and found to be applicable with several different NMs [6,26,36].

## 7. Biomarkers for Genotoxicity

Genotoxicity biomarkers include a battery of assays that cover DNA damage, gene mutations and chromosomal damage as sensitive genotoxicity endpoints. In spite of the limited data and the lack of information concerning NM safety, the number of produced NMs is in constant increase: thus in several past and on-going European projects, such as NanoTEST, NanoGENOTOX, NanoReg, HISENTS, RiskGONE and others, efforts have been made to understand the NMs mode of action and to develop or adapt validated OECD test methods used on chemicals for use on NMs. The biomarkers of genotoxicity have been extensively applied to nanotoxicology risk assessment.

For the risk assessment of NMs in vitro, two test strategy is recommended: (a) mammalian gene mutation test, and (b) test for chromosomal damage.

### 7.1. Mammalian Gene Mutation Test

The most commonly used assays for gene mutation of NMs are based on Tk (thymidine kinase) or Hprt (hypoxanthine phosphoribosyltransferase) genes as described in OECD Test Guidelines (TGs) (OECD TG 476, 2015 and OECD 490, 2015) [114,115], with small adaptations especially regarding NM characterization and exposure [41]. V79 cells were used for detecting Hprt gene mutants after exposure with silver, titanium dioxide, carbon nanotubes, and other NMs [13,31,32,33,116]. The mouse lymphoma (MLA) assay detects a wide spectrum of genetic damage, including gene deletion, as well as epigenetic silencing of the functional Tk allele due to promoter hypermethylation [117]. Several NMs, such as different shapes of silver and iron oxide, were investigated with this assay [66]. 

### 7.2. Chomosomal Damage

Clastogenicity and aneugenicity are important genotoxicity endpoints that identify agents causing structural chromosome or chromatid breaks, dicentrics and other abnormal chromosomes, including loss of chromosomes. These could be detected by chromosomal aberration tests. As this test is time consuming, the most common test to detect chromosomal damage is the in vitro micronucleus assay that detects micronuclei in the cytoplasm of interphase cells [118]. Micronuclei are formed from chromosome, chromatid fragments or whole chromosomes that lag behind in cell division forming single or multiple micronuclei in the cytoplasm. The micronucleus assay detects both structural chromosome damage (clastogenic effect) as well as numerical chromosome alterations (aneugenic effect) [119]. They can be measured by visual or automated scoring after staining slides. The OECD TG 487 (2016) [120] describes the cytokinesis-block micronucleus (CBMN) assay in different cell models, including human or other mammalian cells and cell lines. Cytochalasin B is an agent that is used to block cytokinesis, and thus to prevent separation of daughter cells after mitosis, leading to the formation of binucleated cells. This assay is often used for NM testing; however, exposure with NMs should occur before cytochalasin B is added to allow uptake of NMs by cells [15,31,121].

### 7.3. DNA Damage

Detection of DNA damage (single and double strand breaks and specific DNA lesions) belongs to the group of so-called indicative tests that are also used in hazard assessment of NMs [16,122,123]. DNA strand breaks can be evaluated by the comet assay which is the method of choice for measuring DNA damage in cellular DNA [124]. This assay (also known as single cell gel electrophoresis) is a simple, robust, reliable and user-friendly method, widely used for many years for genotoxicity testing of chemicals, and it is the most commonly applied method to test genotoxicity of NMs [17]. The high sensitivity of the assay, detecting from about 100 up to several thousand breaks per cell, allows detection also of weak genotoxic agents. However, the high sensitivity of the assay may contribute to variability, so to ensure high reproducibility of results, it is important to keep standardized conditions and a constant experimental design.

While the basic comet assay detects strand breaks, a common modification by incorporating digestion with a lesion-specific endonuclease after the lysis step allows detection of damaged bases. Formamidopyrimidine DNA glycosylase (Fpg) has been particularly useful; its primary substrate is the oxidized base 8-oxoGua, and it has therefore been employed to measure the effects of oxidative stress on DNA [125].

DNA double strand breaks (DSBs) and DNA damage foci are DNA damage response biomarkers [126]; local accumulations or modifications of DNA damage response proteins that form at the sites of DNA DSBs can be visualized through microscopic imaging following immunocyto- or -histochemical detection or fluorescent protein tagging [127]. DSBs, among the most severe forms of DNA damage, are triggered by various genotoxic insults and induced directly by a number of physical and chemical agents including NMs [128]. γH2AX is one of the DNA damage response proteins that accumulate and/or are modified in the vicinity of a chromosomal DNA DSB to form microscopically visible, subnuclear foci, contributing to their repair [127]. A number of studies have proposed C-termini phosphorylated histone protein, γH2AX, as a potential biomarker of DNA DSBs caused by genotoxicants [67,129,130,131]. This method is widely used in different fields including in vitro toxicology testing of environmental pollutants [132]. Initially this biomarker was used for identifying ionizing radiation effects [133]. In the last years γH2AX phosphorylation technique has been used in several studies to measure DSB caused by different NMs [15] for instance carbon nanotubes [134,135], zinc oxide [136], gold [137], silica [138], polystyrene [139] and titanium dioxide NMs [50] showing that this technique can be a useful tool to assess the genotoxic potential of NMs [15].

## 8. Carcinogenicity

Crucial for safety assessment of chemicals and NMs is their carcinogenic potential. The observation that a compound is genotoxic implies that it may be potentially carcinogenic. An additional aspect concerning carcinogenicity is the existence of non-genotoxic carcinogens, which induce their effect through secondary mechanisms, such as oxidative stress or other inflammatory responses [140,141,142]. Until recently, the only recognized test for carcinogenicity was by animal testing, such as the 2-year rodent assay. As an ethical response to the importance of reducing the use of animals in safety assessment of chemicals, tests have been developed as alternative methods for detection of carcinogenic potential. An additional important aspect, particularly due to the large numbers of new chemicals and engineered NMs, is the high cost attributed to the animal testing. A ban on in vivo testing of cosmetics has also increased dependence on in vitro tests.

The cell transformation assay (CTA) is an in vitro approach that makes us of the phenotypic transformation of cells as a marker of carcinogenicity [143,144]. Cells transformed in vitro have been shown to induce tumors when injected into immunosuppressed animals. Cells used in CTA are often derived from rodent embryos, such as the Syrian hamster embryo (SHE), mouse BALBc 3T3, Bhas42 and C3H/10T cells. The CTA assays have been met with some reservations and are still not accepted for regulatory purposes. The main argument has been the lack of understanding of the mechanisms behind the transformation in addition to the subjective nature of the assessment of the transformations. A previous study on the Syrian hamster embryo cell transformation assay also reported a high rate of false positives and limitations in the ability to distinguish between rodent and human carcinogens [145]. Recent works have, however, shown that cell transformation assays are useful tools to predict carcinogens [146]. In particular, the CTAs can be useful to identify non-genotoxic carcinogens and should be included as an integral part of a battery of in vitro tests in order to predict carcinogenic potential of chemicals and also NMs [147]. Fontana et al. investigated four different amorphous non-genotoxic silica particles with the CTA and indicated one non-genotoxic carcinogenic out of two pyrogenic and two precipitated silica materials [147].

## 9. New Endpoints of Genotoxicity

Among new endpoints in the study of NM genotoxicity, gene expression and epigenetics have to be taken into account. NM exposure has been reported to induce de-regulation of genes involved in several biological processes, including the DNA damage response and DNA repair, cell cycle progression, oxidative stress and inflammatory responses [148,149,150,151,152,153,154,155]. It is relevant to point out that many of these processes have been shown to lead to secondary genotoxicity [156]. Changes in the regulation of gene expression can occur in response to molecular signaling activated within or between the cells after interaction with chemicals and/or NMs.

Another important class of mechanisms affecting gene expression involves epigenetics, i.e., the study of molecules and mechanisms that can perpetuate alternative gene activity states in the context of the same DNA sequence [157]. During the last decade, epigenetics has become one of the most important areas in the study of biological sciences. Some epigenetic responses have been suggested as possible biomarkers of exposure or disease risk, although the mechanistic links between the endpoints and the outcomes still need to be discovered [158]. Epigenetic responses include, among others, DNA methylation, non-coding small single-stranded RNAs termed microRNAs (miRNAs) and histone modifications. Several metallic, non-metallic and carbon-based NMs have been reported to affect epigenetic mechanisms [53,54,56]. DNA methylation is an important mechanism for the maintenance of cell- and tissue-specific gene expression. The methylated status of the DNA is defined by the addition of a methyl group to the fifth position of the cytosine residue in the dinucleotide sequence CpG [158]. Global hypomethylation has been linked to chromosome instability and oncogenesis, while changes in the methylation status of gene promoter regions entail an alteration of the expression of the associated gene. Changes in the methylation of the DNA, both global and gene-specific, have been reported as an effect of exposure to diverse NMs including silver, silicon dioxide, and silica and carbon nanotubes [52,54,57]. miRNAs are involved in the regulation of gene expression at post-transcriptional level, by affecting the stability of mRNA, or targeting them for degradation. Altered miRNA expression has been related to pathologies including cancer, but also cardiovascular, developmental and neurological diseases. Changes in miRNA expression induced by gold, titanium dioxide and iron nanoparticle exposure have been repeatedly reported in in vitro and in vivo studies [155,156]. Histone modifications include several mechanisms, e.g., acetylation, methylation of lysine residues, phosphorylation, ubiquitination and ATP-ribosylation. These alterations result in chromatin remodeling that consequently influences gene transcription. This is the least studied and understood mechanism in epigenetics, although some studies have reported histone interactions with NMs, such as cadmium telluride quantum dots, soft NMs used as scaffolds for biological and medical applications, and gold NMs [156].

Although genetic and epigenetic changes induced by exposure to NMs have been reported, the causal relationship with the onset of diseases is still poorly understood. There is a need for mechanistic studies associating the molecular responses with the functional effects, e.g., linking the alterations of gene expression with cellular behavior, such as cell migration and invasion, and DNA damage repair capacity. In vitro research on NMs and epigenetics has mainly involved short-term studies [158,159]. Long-term studies with chronic exposures to low doses of NMs are needed to better mimic real human exposure, and to clarify genetic and epigenetic modifications.

Standard molecular biology techniques such as quantitative real time PCR (qPCR), sodium bisulphite sequencing, and pyrosequencing are commonly used to investigate DNA methylation and miRNA expression. These are relevant methods in the study of molecular mechanisms underlying the functional effects. They can for example be coupled with other techniques and genome editing methods such as small interfering RNA (siRNA) or the most recent CRISPR-cas9 to investigate the role of specific genes by selectively altering their expression. However, they have limitations in terms of time, and being labor-intensive and low-throughput [154], although methods development such as high throughput real-time PCR platforms may provide promising systems for the simultaneous processing of large numbers of samples [160]. Besides, a modified version of the comet assay is under development for high throughput screening (HTS) of DNA methylation alterations, and has the potential for testing NMs [161].

## 10. High Throughput Methods

HTS methods are defined as the use of automated tools to facilitate rapid execution of a large number and variety of biological assays that may include several test substances in each assay [162]. 134 publications have been found applying the keywords “genotoxicity & high throughput“ [163,164,165,166,167,168], although only 14 of them related to NMs (Table 1).

In a recent publication developed under EC FP7 NanoREG project, several scientists agreed that the rapid growth of NM production, and the current low throughput, time consuming and laborious approaches for evaluating genotoxicity of the NMs should lead to the development of rapid, efficient and high throughput genotoxicity testing strategies for safety/risk assessment of NMs [15]. HTS methods are needed to allow toxicity testing of large numbers of materials in a timely manner and with savings in terms of labor costs [169].

In a recent review, several existing genotoxicity testing methods which are amenable to HTS/high content screening (HCS) approaches were identified [15]. HTS was introduced in the pharmaceutical and chemical industries as a rapid way of evaluating unwanted effects of novel compounds. HTS in vitro facilitates the hazard ranking of NMs through the generation of a database with all reported effects on biological and environmental systems; thus novel NMs can be prioritized for in vivo testing [170]. Ideally, this screening should allow for mechanistic profiling to better inform on hazard identification and to improve risk assessment strategies [171].

Regulatory in vitro genotoxicity testing exhibits shortcomings in specificity and mode of action (MoA) information [172]. Examples of HTS methods for genotoxicity testing of NMs are described in the following:

### 10.1. Comet Assay

Recent miniaturized versions of the assay- with 12 mini-gels per slide [173], 96 mini-gels on a GelBond film [174,175] a special 96-well multichamber plate (MCP) [176] -allow increased throughput and analysis of numerous types or modifications of NMs in a time- and cost-effective manner. Numerous studies have been published to date on genotoxicity of NMs with the semi-HTP version of the comet assay [53,177,178,179]. To improve the very time-consuming DNA damage evaluation by manual microscopic fluorescence-analysis, several automatic systems for image analysis have been developed to help increase the throughput of the assay for high screening capacity; for instance the fully automated slide-scanning platform Metafer and the MetaCyte CometScan software. Using such automated evaluating systems, the analysis duration is reduced from hour-range to minute-range. Versatile systems conveniently automate a wide area of image analysis applications in microscopy for life sciences and can be adapted to other assays when needed.

### 10.2. In Vitro Micronucleus Assay

Different approaches have been proposed until now to increase the speed of the micronucleus assay in vitro. Classically, the long and tedious visual scoring of slides has been changed by using automated platforms scoring different set of slides in each run [50,60,72,73,74]. The micronucleus assay using HTS/HCS platforms has been widely used in NM research; it requires less test material than conventional test methods, and has a greater compatibility with high throughput screening instrumentation [75,76,77,78,180,181,182]. The standard protocol involves (1) the lysis of membranes by a non-ionic detergent; (2) the use of one or more nucleic acid dyes that can permit discrimination between the liberated nuclei and micronuclei, according to their DNA-dye-associated fluorescence intensities. A high throughput change in the micronucleus assay includes the use of flow cytometry for the separation of micronuclei and nuclei [183,184,185,186,187]. Further modifications use 96-well plates in conjunction with a robotic auto-sampling device. Different commercial automatic scoring devices are now available [178].

### 10.3. γH2AX Assay

Various computer-based approaches have developed the conventional γH2AX assay into a highly efficient technique, which is therefore also suitable for high throughput [82,83]. In addition, this second generation of the γH2AX assay allows the analysis of further parameters in a cell population. Supported by image analysis software tools, foci are identified and quantitative foci parameters are calculated [188,189,190,191,192]. The successful application of this method with iron oxide NMs has been demonstrated by Harris et al. [193] analyzing the H2AX phosphorylation by applying a high content in silico platform.

### 10.4. ToxTracker Assay

The ToxTracker assay (mechanism-based reporter assay based on embryonic stem cells that uses GFP-tagged biomarkers for detecting DNA damage, oxidative stress and general cellular stress) is a tool that could prove useful in the field of NM toxicology allowing for high throughput screening [18,194].

For an even more high throughput approach, (epi-)genome wide analyses, e.g., based on microarray technology (gene or miRNA microarrays), and next generation sequencing such as massive parallel sequencing, allow investigation of changes across the whole (epi-)genome, and can be used, e.g., to screen NM exposure effects on gene expression and epigenetic endpoints such as miRNA alterations. Besides the higher costs of these techniques, difficulties include analysis of the huge amount of data produced, and the evaluation of confounding factors. Powerful bioinformatics tools are needed for data analysis and integration, but genome-wide investigations on the epigenome and transcriptome, coupled with statistical modeling are a relevant approach for the investigation of associations and causal relationships between NM exposure, molecular effects and adverse health outcomes [154].

## 11. New Advanced In Vitro Models (3D, Organ-on-a-Chip)

The development of advanced in vitro models for nanotoxicity assessment is greatly encouraged by the principles of 3Rs, which are embedded in international legislation and regulations on the use of animals for scientific purposes [43]. The goal of 3Rs is to avoid animal experiments whenever it is possible (replace), to limit the number of animals (reduce) and their potential suffering to a minimum (refine). New advanced models such as three-dimensional (3D) in vitro models or organ-on-a-chip technologies (OOC) have the potential to serve as alternative methods to replace animal testing and close the gap between in vivo and two-dimensional (2D) in vitro models [195].

When comparing complex organs with cells cultured in standard 2D monolayers, 2D cultures do not have the ability to represent the functionality of an organ, whereas cells cultured in 3D resemble the organ structure better, due to their more “in vivo-like” behavior for key parameters such as cell viability, proliferation, differentiation, morphology, gene and protein expression and function [195,196]. For genotoxicity assessment, robust protocols for 3D models have been established for skin, airways and liver tissue equivalents [10,197]. Many of the 3D cell culture systems applied in genotoxicity testing (of NMs) have been spheroids, such as liver spheroids constructed from primary hepatocytes, HepG2 hepatocellular carcinoma cells or the HepaRG cell line applied to the comet assay [14,31,34,198,199] and micronucleus assay [200]. Human 3D airway models usually consist of a functional and differentiated respiratory epithelium with cilia and mucus and are cultured on the physiological relevant air-liquid-interface (ALI) [19]. The comet assay has been established with commercially available human reconstructed 3D airway models (MucilAir™, Epithelix and EpiAirway, MatTek) as well as a model consisting of the bronchial epithelial cell line BEAS2B and tumor lung epithelial cell line A549 [4,93]. These 3D models for liver and airways show great promise, but are at an early stage of development and need to be further improved for genotoxicity assessment of NMs. For human skin models, two methods have been developed and used for genotoxicity assessment: the reconstructed skin micronucleus test (RSMN) and the reconstructed skin comet assay (RS comet assay). Reconstructed skin tissues are commercially available (EpiDerm™, Phenion^®^ FT, EpiSkin™) and have been shown to have the same metabolic properties as native skin [94,95]. Wills et al. [32] performed the RSMN with silica nanoparticles and the EpiDerm™ model and came to the conclusion that robust exposure characterization and uptake assessment methods are crucial to interpret nano(geno)toxicity studies successfully. However, 3D tissue-based assays definitely provide a more realistic test system to study the genotoxic potential of NMs, compared to 2D test systems [10,32,34].

Even advanced 3D tissue models fail to mimic an organ in some aspects such as spatiotemporal gradients of chemicals and nutrients and mechano-stimulated environments. The incorporation of microfluidic networks in 3D models constitutes the most recent innovation in microfluidics, called organ-on-a-chip technologies (OOC). OOC models are aimed at modeling in vitro the micro-physiological conditions prevalent in the body as closely as possible, thus avoiding the typical disadvantages of conventional cell models [201]. A major challenge in the cultivation of complex, 3D organ models under physiologically relevant conditions is the maintenance of their function over a long period of time [195]. Bhatia and Ingber [202] described OOCs as systems for the cultivation of living cells in continuously perfused chambers to model physiological functions. OOC have been developed for nearly every organ of the human body for different applications, particularly for drug testing approaches [203]. Besides mimicking one organ under physiological conditions, chip-based in vitro models mimicking organ–organ interactions have also been developed, for example the intestine-liver interaction [204,205]. Additionally, chip-based multi-organ in vitro models are described in literature, using primary cells or cell lines as 2D culture or 3D spheroids to mimic the organs and their interactions on chip [206,207,208,209,210].

For nanotoxicity assessment, a placenta-on-a-chip [211] and a lung-on-a-chip [212] have recently been published, though neither included the endpoint of genotoxicity [107,108]. Researchers developed microfabricated chip technologies for the screening of cytotoxicity and genotoxicity of NMs [213,214,215]. Vecchio et al. coupled the cytokinesis block micronucleus assay with micro-array based cell sorting to analyze the genotoxic effect of NMs on human primary lymphocyte subtypes [215]. In the same year, CometChip Technology was published [123]. The widely used comet assay has been transferred to a microarray-based approach, a high throughput platform, which helps to overcome the limitations of the traditional performed comet assay such as low-throughput and poor reproducibility [123]. Thus, chip technologies can help to transfer important methods to a high throughput level [123] and to combine 3D models with more physiologic features.

## 12. Conclusions and Future Perspectives

Due to constantly growing commercial applications and their presence in the majority of consumer products, NMs are being extensively investigated for their safety. A main concern with NM exposure is their genotoxic potential. Therefore, the study of mechanisms potentially leading to genotoxicity is crucially important. Besides the general mechanisms of genotoxicity (primary direct genotoxicity and secondary genotoxicity) and their verification processes, we have described the additional studies which are needed for a correct implementation of genotoxicity studies. Before genotoxicity testing, it is necessary to determine the LC_50_ (lethal concentration) in order to define the appropriate dose range for genotoxicity studies. Furthermore, for the interpretation of the results a characterization of the NMs under physiological conditions is essential for the correct interpretation of the biologic data. Due to the limited data concerning NMs safety and the increasing number of yearly produced NMs, different European projects, such as NanoTEST, NanoGENOTOX, NanoReg, HISENTS, RiskGONE and others, made efforts to understand the mode of action of NMs and to develop, or adapt validated OECD chemical test methods for use on NMs. Genotoxicity biomarkers include a battery of assays that were discussed in this chapter. We described the main endpoints applied in standard in vitro models as well as new advanced 3D models that are closer to the in vivo situation. New genotoxicity endpoints were discussed, including genetic and epigenetic changes. We have discussed 3D models and their application for genotoxicity studies as well as the state-of-the-art on adaptation of genotoxicity assays to HTS technologies. The development of advanced in vitro models for nanotoxicity assessment is greatly encouraged by the principles of 3Rs (replace, reduce, refine), which are embedded in international legislation and regulations on the use of animals for scientific purposes. Additionally, their incorporation in microfluidic systems (organ-on-a-chip technologies) are very challenging approaches. Existing approaches of organ-on-a-chip and HTS methods are presented and discussed, such as the use of automated tools to facilitate rapid execution of a large number of comet assays, in vitro micronucleus assays or γH2AX assays.

The need for HTS and rapid automated procedures to perform genotoxicity studies is growing, both in the pharmaceutical and in the chemical industry for approval of new nanoscale materials. A high number of international experts are working on the optimization of the procedures described in this chapter, with the aim of approving the new advanced cell models as new animal replacement models and including the automated culture and analysis procedures in new OECD Test Guidelines.

## Figures and Tables

**Table 1:** Number of publications during the last 20 years in the field of advanced in vitro 3D models, organ-on-chip and high throughput methods suitable for detection of genotoxicity of nanomaterials. An electronic literature search was conducted through PubMed databases to identify articles dealing with genotoxicity of nanomaterials and advanced in vitro models and high throughput methods from year 2000–2020 (August). The listed keyword combinations were used.

Keywords of Literature Search	Number of Publications Per Time Period		
	2000–2020	2010–2020	2019–2020
genotoxicity and 3D models in vitro	59	47	23
genotoxicity and advanced in vitro model	73	63	23
genotoxicity and & high throughput	134	120	32
genotoxicity and organ on chip	7	7 *	2
genotoxicity and 3D models & nanoparticles	7	7 **	1
genotoxicity and 3D models & nanomaterials	11	11 ***	5
genotoxicity and high throughput & nanoparticles	6	6 ***	4
genotoxicity and high throughput & nanomaterials	8	8 ***	4

* first paper published in 2013; ** first paper published in 2014; *** first paper published in 2011.

**Table 2:** Overview of the in vitro studies published in the field genotoxicity of nanomaterials in combination with a 3D model, organ-on-chip and high throughput methods. All publications found via PubMed literature search to the following keyword combinations are listed in this table. “genotoxicity & 3D models & nanoparticles”, “genotoxicity & 3D models & nanomaterials” and “genotoxicity & organ on chip”. ECL: electrochemiluminescent (no nanomaterial tested); CFA: colony forming assay; MNA: micronucleus assay; IFL: immune fluorescence; SWCNT: single walled carbon nanotube; MWCNT: multi walled carbon nanotube; UFCB: ultrafine carbon black; ASB: crocidolite asbestos; ZnO: zinc oxide; TiO_2_: titanium dioxide; Ag: silver; LD: live/dead; n.sp.: not specified; LDH: lactate dehydrogenase; ATP: adenosine tri-phosphate; SiO_2_: silica; ZrO_2_: zirconium dioxide; TEM: transmission electron microscopy; RPD: relative population doubling analysis; NM: nanomaterial.

Human Cell Origin	Cell Model	Culture Dimension	Endpoints	Test Methods	NM Tested	Year	Ref
*Nasal mucosa*	mini organ culture	3D	cytotoxicity, genotoxicity	comet assay, caspase–3 ELISA, ROS assay, TEM	ZnO	2011	[44]
*lung*	EpiAirway™	3D	cytotoxicity, genotoxicity	comet assay, LDH assay, ATP assay	Ag, SiO_2_, ZrO_2_	2017	[33]
*lung*	small airway epithelium	2D	cytotoxicity, genotoxicity	apoptosis assay, CFA, p53 downregulation, IFL, γH2AX assay, spheroid formation assay	SWCNT, MWCNT, UFCB, ASB	2019	[39]
*skin*	EpiDerm™, TK6	2D & 3D	cytotoxicity, genotoxicity	MNA, TEM, RPD analysis	SiO_2_	2016	[32]
*liver*	HepG2	2D & 3D	cytotoxicity, genotoxicity	comet assay, LD staining, alamarBlue™ assay	Ag, ZnO, TiO_2_	2020	[34]
*liver*	HepG2	3D	cytotoxicity, genotoxicity, liver functionality	MNA, cytokine secretion	Ag, TiO_2_	2020	[38]
*liver*	HepG2, HepaRG	3D	cytotoxicity, genotoxicity, liver functionality	MNA, TB assay, albumin level, urea level	ZnO	2020	[37]
*liver*	micro tissue	3D	cytotoxicity, genotoxicity, liver functionality	comet assay, LD staining, adenylate kinase assay, cytokine secretion, comet assay, albumin ELISA, CYP3A4 activity, lipid peroxidation assay	Ag, ZnO, MWCNT, TiO_2_	2014	[31]
*liver, blood, breast*	HepG2, TK6, MCF7	2D	genotoxicity	comet assay, Epi-comet assay	-	2017	[24]
*liver lung kidney, intestine*	n. sp.	2D	genotoxicity	ECL fluidic chip LC-MS/MS	-	2015	[25]
